# A New Policy on Tobacco Papers

**DOI:** 10.1371/journal.pmed.1000237

**Published:** 2010-02-23

**Authors:** The PLoS Medicine Editors

## Abstract

In this month's editorial, the *PLoS Medicine* editors announce that they will no longer consider papers for which support - in whole or in part - for the study or the researchers comes from a tobacco company.

This past month *PLoS Medicine* published two original analyses on smoking, the single greatest preventable risk for poor health and death in the developed world, and an increasingly important risk factor in the developing world. The first study, using internal tobacco company documents unsealed through litigation, provides further evidence of the already well-documented strategy of deception used by the tobacco industry to further its commercial activities. The second study shows the ways in which the tobacco control agenda is distorted by the increasing medicalization of smoking cessation.

In the first paper, Katherine Smith and colleagues report how British American Tobacco (BAT), the world's second largest tobacco transnational, strategically influenced the European Union's framework for evaluating policy options, leading to the acceptance of an agenda that emphasizes business interests over public health [1]. The researchers examined over 700 internal BAT documents that contain information on the company's attempts to influence European regulatory reform and conducted interviews with European policymakers and lobbyists. Their analyses show that BAT created a policy network of representatives from many corporations involved in marketing products that are damaging to public health and the environment, which then successfully campaigned to have specific changes made to the EU Treaty that allowed policymakers to reduce the regulatory burden on businesses. These changes therefore set up conditions that may allow future European policy to favor businesses rather than the health of citizens.

In the second paper, Simon Chapman and Ross MacKenzie critique the dominant messages about smoking cessation contained in most tobacco control campaigns, which emphasize that serious attempts at quitting smoking must be pharmacologically or professionally mediated [2]. This has led to the medicalization of smoking cessation. In fact, argue the authors, there is good evidence that the most successful methods used by most ex-smokers are quitting “cold turkey” or reducing then quitting. The medicalization of smoking cessation is propped up by the extent and influence of pharmaceutical support for cessation intervention studies, say the authors. They cite a recent review of randomized controlled trials of nicotine replacement therapy that found that 51% of industry-funded trials reported significant cessation effects, while only 22% of non-industry trials did [3].

This month also marks the implementation of a new policy on tobacco papers at *PLoS Medicine*.

While we continue to be interested in analyses of ways of reducing tobacco use, we will no longer be considering papers where support, in whole or in part, for the study or the researchers comes from a tobacco company. As a medical journal we do this for two reasons. First, tobacco is indisputably bad for health. Half of all smokers will die of tobacco use [4]. Unlike the food and pharmaceutical industries, the business of tobacco involves selling a product for which there is no possible health benefit. Tobacco interests in research cannot have a health aim—if they did, tobacco companies would be better off shutting down business—and therefore health research sponsored by tobacco companies is essentially advertising. Publication is part of tobacco company marketing, and we believe it would be irresponsible to act as part of the machinery that enhances the reputation of an industry producing health-harming products.

Second, we remain concerned about the industry's long-standing attempts to distort the science of and deflect attention away from the harmful effects of smoking. That the tobacco industry has behaved disreputably—denying the harms of its products, campaigning against smoking bans, marketing to young people, and hiring public relations firms, consultants, and front groups to enhance the public credibility of their work—is well documented. There is no reason to believe that these direct assaults on human health will not continue, and we do not wish to provide a forum for companies' attempts to manipulate the science on tobacco's harms.

Furthermore, the business model used to support our open access publishing (the research funder covers publication costs, unless the author requests a waiver) means we would essentially be accepting money from the tobacco industry by publishing their papers. This is unacceptable to the editorial team of *PLoS Medicine*.

Our new policy may be criticized as moralistic, unscientific, and against transparency. Indeed, the leading tobacco control journal (*Tobacco Control*) does not ban tobacco industry–funded research, for two reasons: it wishes to avoid being labeled as biased by the industry, and it does not think it sensible to single out tobacco when the food and drug industries also have deeply vested and conflicted interests in the research supporting their corporate agendas [5]. Journals such as *BMJ* have also rejected a ban on research papers from authors funded by the tobacco industry, citing such a move as a form of unacceptable censorship and instead managing the potential competing interests as it would all papers [6]. Ten years ago, one of us (GY) argued for the *BMJ* position [7], but has changed his view over the last decade in the face of increasing evidence of the tobacco industry's distortion of science.

But other journals such as those of the American Thoracic Society do have such policies—since 1995 they have not accepted any medical research that is funded by the tobacco industry, and they explicitly do so on moral and ethical grounds [8].

Like the two other PLoS journals that have recently adopted this policy, *PLoS Biology* and *PLoS ONE*, we feel that any potential criticisms and risks are preferable to supporting the tobacco industry's efforts to deflect attention from the harms of its products. It is the case that we do not receive many tobacco industry sponsored papers—*PLoS Medicine* has published none since our inception in 2004 and *PLoS ONE* only two—and we have made previous editorial judgments on papers that might be favorable to the tobacco industry agenda on a case-by-case basis [9]. We wish now to formalize our policy effective immediately.
